# Extraction of high-value compounds from *Theobroma grandiflorum* (cupuassu) seed shells using pressurized liquid extraction with NADES: a green chemistry approach

**DOI:** 10.1007/s00216-026-06318-3

**Published:** 2026-01-29

**Authors:** Paulo Natan Alves dos Santos, Marcos Levi Cazaes Machado dos Reis, Bruna Louíse de Moura Pita, Fabio de Souza Dias, Alini Tinoco Fricks, Elina Bastos Caramão

**Affiliations:** 1 Rede Nordeste de Biotecnologia, Universidade Federal de Sergipe, São Cristóvão, SE 49107-230 Brazil; 2 Programa de Pós-Graduação Em Química, Universidade Federal da Bahia, Salvador, BA 40170-115 Brazil; 3 Faculdade de Farmácia, Universidade Federal da Bahia, Salvador, BA 40170-115 Brazil; 4 Departamento de Análise Bromatológica, Programa de Pós-Graduação Em Ciência Dos Alimentos (PGALI), Faculdade de Farmácia, Universidade Federal da Bahia, Salvador, 40170-115 Brazil; 5 Instituto Nacional de Ciência E Tecnologia, Energia E Ambiente (INCT E&A), Salvador, BA Brazil

**Keywords:** Cupuassu seed shell, Fatty acids, Theobromine, Energized dispersive guided extraction, Natural deep eutectic solvents, Green chemistry

## Abstract

The species of *Theobroma* grandiflorum, commonly known as cupuassu, is widely used in the production of various cosmetic and food products. However, cupuassu seed shell (CSS), a major agro-industrial residue generated during the processing of its seeds, remains largely underexplored. In this study, a dual-extraction strategy was developed to valorize CSS using green chemistry principles, emphasizing waste recovery, reduced solvent consumption, and lower environmental impact. This methodology was applied to the extraction of theobromine, a methylxanthine of growing interest in the development of nutraceuticals and functional foods. In the first step, lipids were extracted using the energized dispersive guided extraction (EDGE) system, an automated and sustainable alternative to conventional methods. Fatty acids analysis revealed an oil profile like that of commercial cupuassu seed oil, reinforcing the potential of CSS oil as a functional ingredient in food, pharmaceutical, or cosmetic applications. In the second step, defatted CSS solid residue was subjected to theobromine extraction using the same EDGE system, this time employing a natural deep eutectic solvent (NADES) composed of choline chloride and glycerol (1:3). Extraction conditions were optimized using a BBD (Box–Behnken Design), with the best results achieved at 150 °C, 15 min, and 80% NADES, yielding 0.62 mg g^−1^ in dry basis (d.b.) of theobromine. This study demonstrates a sustainable and efficient approach to convert a low-value byproduct into high-value bio-based compounds, highlighting the versatility of the EDGE system and reinforcing the principles of a circular economy and green analytical chemistry.

**Graphical abstract:**

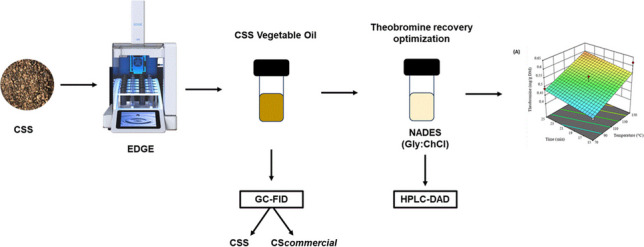

## Introduction

Native to the Amazon rainforest, *Theobroma grandiflorum* (commonly known as cupuassu), has been traditionally used by indigenous populations for both nutritional and medicinal purposes. Its cultural significance is also reflected in the name of its genus, “Theobroma,” derived from Greek and meaning “food of the gods” [1]. This species is used to produce a wide range of products, such as beverages, creams, ice cream, yogurt, compotes, jams, jellies, puddings, a chocolate-like product known as “cupulate” [2], with studies being conducted to explore its pulp [3], shell [4] and seeds [5].

Among the valuable components found in cupuassu seeds are fats and methylxanthines. The fat, similar to cocoa butter, has a smooth texture and melts below body temperature, making it promising for food and cosmetics, with a market projected to reach USD 62 million by 2030 [1, 2]. Theobromine, a methylxanthine highlighted as a mild central nervous system stimulant without addiction risk, as well as its potential cardiovascular and anti-inflammatory benefits [6]. Moreover, despite its potential as a source of fats and theobromine, cupuassu seed shell (CSS), a residual byproduct of cupulate production, remains underexplored in scientific literature.

Although conventional extraction methods are widely used to recover bioactive compounds due to their simplicity and low cost, they rely on harmful solvents, long extraction times, and high temperatures, which may degrade phytochemicals [7]. Thus, novel sustainable extraction techniques, such as Energized Dispersive Guided Extraction (EDGE), which combines dispersive solid-phase extraction (DSPE) with pressurized liquid extraction (PLE), offer an alternative to conventional methods commonly used in analytical chemistry with eco-friendly attributes such as reduced solvent consumption and shorter operation times [8, 9].

Thus, evaluating and minimizing the environmental impact of analytical procedures is essential. Green Analytical Chemistry (GAC) addresses this by promoting eco-friendly practices without compromising result integrity [10]. Driven by global sustainability goals, researchers now seek to optimize entire processes—not just reduce waste or energy use. To guide these efforts, twelve principles of green chemistry were proposed, including waste prevention, the use of renewable materials, and safer chemicals and solvents [11]. In this context, Natural Deep Eutectic Solvents (NADES), firstly proposed by Abbott et al. [12], and prepared from bio-based starting materials (with hydrogen bond donors and acceptors to form a eutectic mixture), have emerged as contestants for green extraction.

Loukri et al. [13] proposed a eutectic mixture based on choline chloride and glycerol as a green and efficient extraction medium for recovering caffeine from coffee pulp, demonstrating its potential for methylxanthine extraction. In this context, the choline chloride–glycerol (Ch:Gly) NADES was selected in the present work due to its biocompatibility, low toxicity, availability of components, and previously reported efficiency in methylxanthine extraction. Furthermore, our research group has recently focused on developing sustainable approaches for methylxanthine recovery using eutectic and conventional solvents.

For instance, Nascimento et al. [14] employed a glycerol–urea mixture under ultrasound-assisted extraction, obtaining methylxanthine-rich extracts suitable for food, beverage, and nutraceutical applications. Nascimento et al. [15] also reported an EDGE procedure based on ethanol–water mixtures to extract caffeine and theobromine from cocoa bean shells. However, the application of eutectic solvents in the EDGE system has not yet been reported, despite their recognized potential for improving extraction efficiency. Thus, the use of the Ch:Gly system was considered a rational choice to evaluate the feasibility of integrating a green, well-documented NADES into the EDGE extraction process, while addressing key challenges related to viscosity, pH, and pressure [12].

To enhance the efficiency of extraction procedures, multivariate optimization techniques such as Response Surface Methodology (RSM) have proven to be powerful tools, enabling the evaluation of interactions between variables and the identification of optimal operational conditions with fewer experiments compared to univariate approaches [16]. In this study, a Box–Behnken design (BBD) was employed to optimize key parameters in theobromine extraction using NADES, aligning with the principles of green analytical development.

Based on this assumption, this study developed a dual-extraction strategy to valorize CSS following green chemistry principles. First, fats were extracted by EDGE and analyzed by gas chromatography coupled to flame ionization detection (GC-FID), comparing their profile to commercial cupuassu seed oil. Then, the degreased residue was used to extract theobromine with a eutectic mixture based on choline chloride:glycerol. Extraction parameters were optimized by RSM using a BBD, and theobromine content was quantified by high-performance liquid chromatography coupled with diode array detection (HPLC-DAD).

## Experimental

### Materials and chemicals

The water used was ultrapure, with a specific resistivity greater than 18 MΩ cm, obtained from a Milli-Q® water purification system (Millipore, Bedford, USA). All solvents were chromatographic grade obtained from J. T. Baker. ZnCl_2_ (analytical purity > 99%). Choline Chloride 98% and glycerin 99% were purchased from Sigma Aldrich. Chromatographic standards were obtained from Supelco (Supelco Park, PA, USA). The gases used for the chromatographic analysis had purities greater than 99.999%, purchased from White Martins (Aracaju, SE).

The commercial oil (from CS) was purchased in a local market. CSS was donated by an organic chocolate factory located in Salvador, Bahia, Brazil, according to the geographic coordinates: latitude − 12°54′13.6728″, − 38°26′34.3926″. It was oven-dried at 40 °C for 72 h, ground using an industrial blender (FAK LAR-4), and subsequently subjected to pre-treatment steps.

### NADES Ch:Gly synthesis

The NADES used in this study was composed of choline chloride as hydrogen bond acceptor (HBA) and glycerol as hydrogen bond donor (HBD) with a molecular ratio of 1:3, and prepared according to Loukri et al. [13], mixing in a heating stirring plate (IKA C-MAG HS10) both components at 600 rpm at a temperature of 70 °C for approximately 1 h, until a transparent mixture was obtained.

### Attenuated total reflectance Fourier transform infrared spectroscopy (ATR-FTIR)

ATR-FT-IR spectra were acquired using a FT-IR Spectrum Two (Perkin-Elmer) spectrometer equipped with attenuated reflectance technology (ATR). For each spectrum, 20 scans were accumulated in the absorbance mode and recorded at 4 cm^−1^ resolution covering a range from 4000 to 400 cm^−1^. The spectrum was collected against a background obtained with a dry and clean cell. Six spectra per sample were recorded and averaged in order to obtain the corresponding spectrum before further pre-processing.

### EDGE procedure

#### Fatty acids extraction

Details on the configuration and use of EDGE system as well as the name of its parts and accessories is described in detail in a previous work [10]. We will use the same nomenclature here. Employing this technique and a solvent commonly used to degrease samples (hexane p.a.), fatty acids from CSS were extracted according to Lucas et al. [8] with brief modifications. Approximately 3.5 g of CSS was used and three 15 min extraction cycles were performed, with 30 mL of hexane each one, at 140 °C. After the pre-treatment step, the fixed oil obtained was subjected to solvent evaporation and stored for further analysis by GC-FID, being compared to a commercial CS oil.

#### Theobromine extraction

Theobromine extraction procedure was conducted using 500 mg of degreased CSS directly weighed into a Q-Cup containing a set of S1 Q-Disc filters and 20 mL of Ch:Gly NADES mixture. These parameters were employed in accordance to Conrado et al. [9]. After each extraction was completed, a washing step was performed by passing 10 mL of the extracting solvent at 70 °C for 30 s in the system, preparing the instrument for the next sample. The extracts were collected and stored at − 8 °C until further analysis.

### Optimization procedure

For EDGE optimization procedure, temperature (°C), heating time (min), and NADES concentration (%) were studied according to the parameters employed by Dos Santos et al. [7] and Loukri et al. [13] with brief modifications. The assays were done in random order, and triplicates of the center point were performed to evaluate experimental error. The analytical response was theobromine content obtained by HPLC-DAD. Experimental data were processed using Design Expert 12 software.

### Chromatographic analysis

#### Saponification and derivatization of total fatty acids

The breakdown of the glycerides present in the vegetable oil was carried out by saponification followed by a methylation using BF_3_ in an alkali solution of methanol (with KOH) [17]. An aliquot of 100 μL of each oil was saponified with 3 mL of potassium hydroxide in methanol (0.5%, w/v) at 100 °C for 15 min in a round-bottom flask. Esterification was performed using 3 mL of boron trifluoride (BF₃) in methanol under the same conditions. Fatty acid methyl esters (FAMEs) were extracted by adding 3 mL of *n*-hexane, followed by 3 mL of saturated sodium chloride solution and manual agitation. After phase separation, 1 mL of the organic phase was collected and analyzed by GC**-**FID.

#### Gas chromatography analysis

Fatty acids present in CSS oil obtained by EDGE extraction procedure and the commercial oil were identified and semi-quantified by gas chromatography coupled to flame ionization detector according to the conditions established by Souza et al. [18]. Samples were analyzed using a GC-FID 2010 apparatus (Shimadzu, Japan) with a SUPELCOWAX-10 column (30 m × 0.25 mm × 0.25 µm. The oven temperature was initially set at 70 °C for 3 min, then increased to 210 °C at a rate of 24 °C/min, followed by an additional increase to 230 °C at a rate of 5 °C/min, where it was maintained for 8 min. Nitrogen was employed as the carrier gas at a rate of 1.7 mL min^−1^. The injector was operated in split mode (1:20) at a temperature of 230 °C, with an injection volume of 1 μL. Injections were performed in triplicate for all samples.

To determine fatty acid content in oil samples, methyl esters present were identified by comparison with the retention times of FAME external standard. A correlation factor was calculated for all methyl esters of fatty acids present in cupuassu seed shell and cupuassu seed oils. The concentrations were expressed as Relative Response Factor percent (RRF %): the ratio between the area of the compound and the area of an internal standard (methyl heptadecanoate) at a concentration of 1000 mL min^−1^.

#### Liquid chromatographic analysis

Theobromine present in the extracts obtained using NADES-EDGE were identified and quantified by liquid chromatography according to the conditions established by Nascimento et al. [15]. Samples were analyzed using an HPLC model LC20AD (Shimadzu, Tokyo, Japan) system consisting of an autosampler and diode array detector (DAD). An RP-C18 column, 4.6 × 150 mm with porosity of 5.0 μm (Phenomenex, Torrance, CA, USA), and a guard column (4.6 mm ID × 12.5 mm) were employed. The oven temperature was maintained at 45 °C. The mobile phase was composed of water–acetic acid (99:1) (Solvent A) and acetonitrile (Solvent B) at a flow rate of 1.0 mL min^−1^. An isocratic mode was employed using 80% solvent A. UV absorbance was monitored from 200 to 400 nm. Identification was performed by comparing the retention time of theobromine present in the extracts with those of its corresponding analytical standard. Theobromine (mg/g dry basis) was quantified at 273 nm based on the integrated peak areas of the samples and standard (Sigma-Aldrich, Berlin, Germany) using external calibration.

The validation parameters (limits of detection; limits of quantification, regression equation, and correlation coefficients and linear range) of the liquid chromatographic method were reported in a previous study developed by Nascimento et al. [14].

## Results and discussion

### Fatty acid characterization

The fats from CSS obtained by EDGE were chemically characterized by GC-FID, and its profile was compared to commercial cupuassu seed oil (CS*commercial*), as shown in Table 1. CSS oil was mainly composed of oleic acid (48.97%), stearic (23.83%), linoleic (9.55%), palmitic (8.62%), and arachidic acids (7.38%), with minor amounts of linolenic (0.38%) and behenic acids (1.27%). Venturini et al. [19] reported that fats extracted from cupuassu seeds are solid lipids at room temperature and contain saturated and unsaturated fatty acids, especially linoleic and palmitic acids. Serra et al. [20] analyzed by GC-FID several Amazonian fruit oils obtained by artisanal cold pressing and reported that cupuassu seed fat is mainly composed of oleic and stearic acids, consistent with this study.

**Table 1:** Chromatographic relative response factor percent (RRF %) for CSS and CS*comercial* fatty acids

Fatty acid	RRF (%) for CSS and CS*comercial* oil	
	CSS	CS*commercial*
C 16:0 (palmitic)	8.62	9.30
C 18:0 (stearic)	23.83	27.13
C 18:1 (oleic)	48.97	47.10
C 18:2 (linoleic)	9.55	7.34
C 18:3 (linolenic)	0.38	0.27
C 20:0 (arachidic)	7.38	7.93
C 22:0 (behenic)	1.27	0.93
Σ SFAS	41.11	45.29
Σ MUFAS	48.97	47.10
Σ PUFAS	9.93	7.61

*SFAS* saturated fatty acids, *MUFAS* monounsaturated fatty acids, *PUFAS* poly-unsaturated fatty acids

**Fig. 1:**
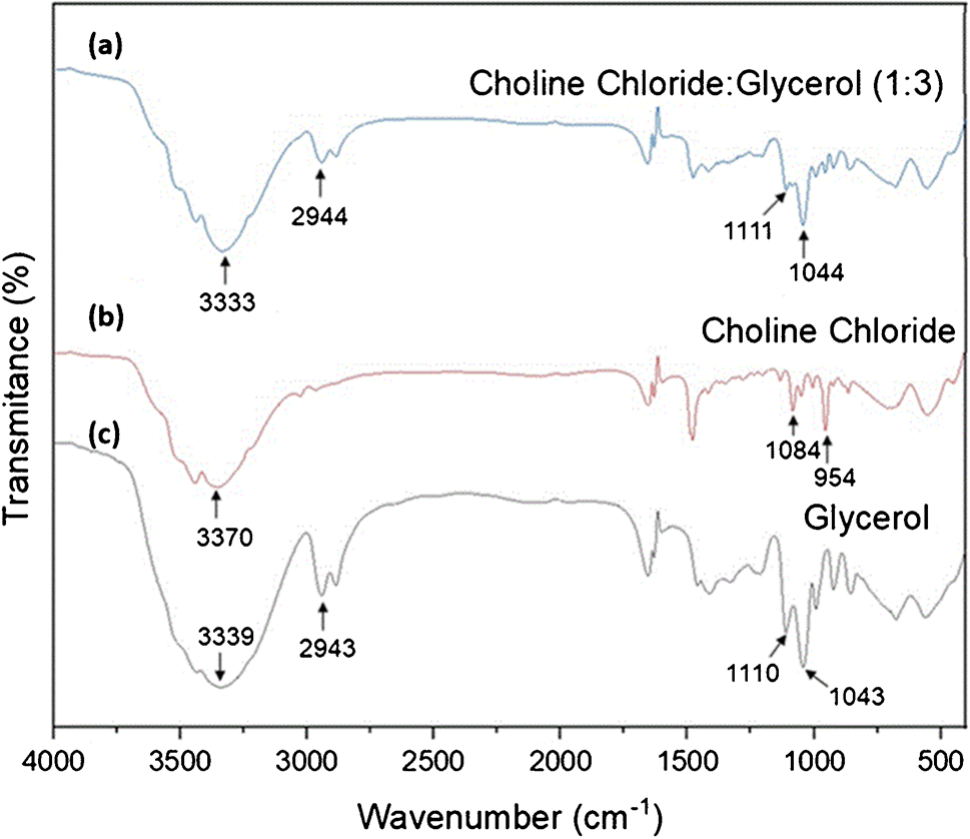
Infrared spectra corresponding to **a** pure Ch:Gly, **b** choline chloride, and **c** glycerol

Although CS*commercial* oil exhibited a similar fatty acid profile, it presented slightly higher contents of palmitic (9.30%) and stearic acids (27.13%), slightly lower oleic (47.10%), and linoleic acids (7.34%), while linolenic (0.27%), arachidic (7.93%), and behenic acids (0.93%) were found at similar concentrations. The chromatographic profile is presented in Fig. 1. According to Wiltshire et al. [21], factors such as genetics, climate, seasonality, and agronomic practices can influence the yield and composition of vegetable oils. These variables likely explain the differences between CSS and CS*commercial* oils. Still, their similar composition highlights the industrial potential of CSS fats and the value of utilizing this underused byproduct to promote economic and sustainable circular practices.

Oleic acid is a monounsaturated fatty acid widely employed in pharmaceutical preparations. Through its binding to circulating lipoproteins, this fatty acid enhances intestinal absorption, cellular uptake, and bioavailability of hydrophobic drugs [22]. Different from other saturated fatty acids, stearic acid does not elevate plasma cholesterol levels, and it has been demonstrated to reduce plasma low-density lipoprotein levels, thereby contributing to a hypocholesterolemia response. Due to its unique biological properties on metabolic systems, recently, stearic acid has emerged as a safer choice which has little effect on major risk factors for the occurrence and pathogenesis of cardiovascular diseases, tumors, diabetes, and even brain neurological diseases [23].

Palmitic acid, a saturated long-chain fatty acid, demonstrates anti-inflammatory activity, metabolic regulatory effects, and displays antitumor properties across various types of tumors. Linoleic acid, a major constituent of various seeds, shows benefits related to reduced cholesterol and triglycerides, anti-inflammatory, cardioprotective, anticancer, and antidiabetic effects in humans [9]. Nevertheless, the fatty acid profile of *T. grandiflorum* seeds is similar to cocoa butter (*T. cacao*), with slight differences, notably in palmitic acid content. Given that *T. grandiflorum* butter and cupulate are commercially available, new industrial applications can be developed to complement or replace traditional *T. cacao* products, enhancing market resilience and economic adaptability during off-seasons [1].

To the best of our knowledge, only two authors have investigated the use of the EDGE system for the extraction of fatty acids from biomass. Lucas et al. [8] were the first to explore this procedure. The authors applied it to spent coffee grounds, silverskin, microalgae, macroalgae, and tobacco biomasses. The analysis by GC/TOFMS allowed the identification of palmitoleic, palmitic, linoleic, elaidic, oleic, linolenic, and stearic acids, resulting in the production of pyrolytic bio-oils with lower fatty acid content, favoring the production of other high-value compounds, suggesting that the EDGE system is suitable for biomass enhancement, especially due to its low solvent consumption and fast extraction time, offering advantages in terms of reproducibility and minimization of human errors when compared to traditional extraction methods such as Soxhlet or ultrasound- assisted extraction.

Conrado et al. [9] applied the same technique to extract lipid fraction from açaí seeds, identifying lauric, myristic, palmitic, stearic, oleic, and linoleic acids by GC/qMS, reporting an oil yield seven times higher than that obtained with the classical Soxhlet method. The method also exhibited low relative standard deviations, ranging from 0.1 to 0.9%, which, according to Dos Santos et al. [7] can be attributed to minimal experimental variation inherent to fully automated procedures compared to more manual approaches. In addition, the present study, also conducted using the EDGE system for the same purpose, required approximately nine times less solvent (45 mL vs. 400 mL) and was nearly five times faster (45 min vs. 200 min).

Nevertheless, further studies are warranted to optimize the degrease of plant matrices, with the goal of producing vegetable oils with comparable chemical composition while minimizing biomass usage, solvent consumption, and extraction time—aligning even more with the principles of green analytical chemistry by prioritizing safer and more sustainable extraction methods.

### NADES characterization

**Fig. 2:**
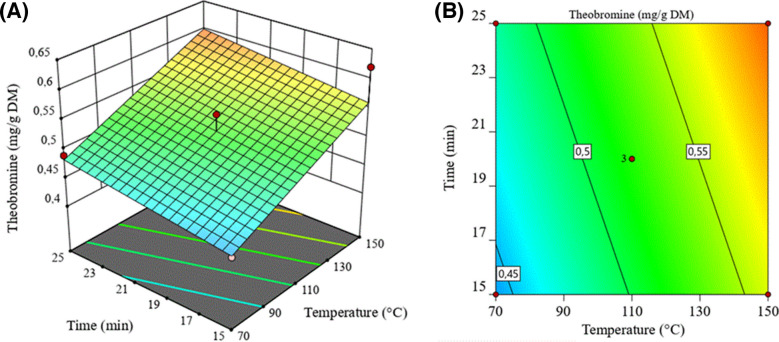
Response surface (**A**) and contour charts (**B**) obtained from Box–Behnken design

The technique of attenuated total reflectance Fourier transform infrared spectroscopy (ATR-FTIR) was used to characterize the synthesized NADES. Spectra were obtained for glycerol, choline chloride, and the NADES. In Fig. 2, characteristic bands of glycerol can be observed, such as a band at 3339 cm⁻^1^, indicative of O–H stretching, and bands at 1110 cm⁻^1^ and 1043 cm⁻^1^ attributed to C-O and C–OH stretching. For choline chloride, the relatively broad peak at 3370 cm^−1^ is the result of O–H stretching vibrations. At 1111 cm^−1^, C-O stretching vibrations can be noticed while peaks at 1044 cm^−1^ can be linked with C–OH bending vibrations.

The NADES shows bands at 3333 cm⁻^1^, 2944 cm⁻^1^, 1111 cm⁻^1^, and 1044 cm⁻^1^, indicating interactions between its components that affect the vibrational frequencies of functional groups. The presence of bands in regions like choline chloride and glycerol suggests that these are components of the solvent. The observed shifts indicate new intermolecular interactions, as predicted by Dai et al. [24] since NADES alter physicochemical properties of individual components, creating more stable and efficient systems based on hydrogen bonding.

### Theobromine extraction: optimization and comparison with other alternatives

To obtain the best experimental conditions, a BBD was applied as a response surface method. A comparison between BBD and other response surface designs (central composite, Doehlert matrix, and three-level full factorial design) has demonstrated that BBD and the Doehlert matrix are slightly more efficient than the central composite design, but much more efficient than the three-level full factorial designs, where the efficiency of one experimental design is defined as the number of coefficients in the estimated model divided by the number of experiments [16].

In addition, this tool provides a comprehensive optimization analysis, highlighting the influence of parameters on the objective function and their interactions, offering a response surface that facilitates the visualization of results derived from the experimental analysis [25]. In this context, temperature, heating time, and NADES concentration were studied according to the matrix presented in Table 2.

**Table 2:** Coded values, real values, and the respective responses to BBD applied in energized dispersive guided extraction

Exp	Temperature, °C	Time, min	NADES concentration, %	Theobromine content, mg g^−1^_d.b_.*
1	(−) 70	(0) 20	(−) 65	0.41
2	(−) 70	(0) 20	(+) 95	0.48
3	(+) 150	(0) 20	(−) 65	0.54
4	(+) 150	(0) 20	(+) 95	0.58
5	(0) 110	(−) 15	(−) 65	0.47
6	(0) 110	(−) 15	(+) 95	0.47
7	(0) 110	(+) 25	(−) 65	0.58
8	(0) 110	(+) 25	(+) 95	0.54
9	(−) 70	(−) 15	(0) 80	0.44
10	(+) 150	(−) 15	(0) 80	0.62
11	(−) 70	(+) 25	(0) 80	0.49
12	(+) 150	(+) 25	(0) 80	0.55
13 (C)**	(0) 110	(0) 20	(0) 80	0.55
14 (C)**	(0) 110	(0) 20	(0) 80	0.55
15 (C)**	(0) 110	(0) 20	(0) 80	0.55

*d.b., dry basis; ** (C) = central point

A linear model was utilized to describe the experimental response surface, and analysis of variance (ANOVA) was employed to assess its statistical significance. The model exhibited a p-value of 0.0063, indicating that it was statistically significant at the 95% confidence level. The F-value of 7.14 further confirmed the adequacy of the model in explaining the response variability. The lack of fit was not significant, with a sum of squares of 0.0161 and a mean square of 0.0018, suggesting that the model fits the experimental data. The obtained linear model is given by Eq. (1):

1 $$Theobromine\;content\;\left(mg\;g^{-1}d.b.\right)=0,5213+0,0587\times A+0,02\times B+0,0088\;\times C$$

where **A** refers to the extraction temperature (°C), **B** to the extraction time (min), and **C** to the concentration of the natural deep eutectic solvent (%).

**Fig. 3:**
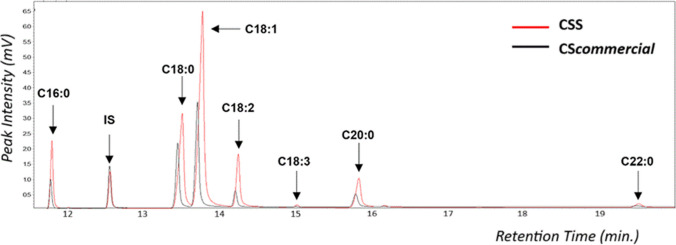
Comparison among the chromatographic profiles obtained by GC-FID of CBS and CS oil. Experimental conditions described in the text

A visual inspection of the response surface could be performed to determine the best operational conditions (Fig. 3) [26]. The highest theobromine content (0.62 mg g⁻^1^ d.b.) was obtained under the conditions of 150 °C, 15 min of heating time, and 80% NADES concentration. These conditions correspond to the maximum region of the response surface, as observed in the contour plots, which visually support the selection of these optimal parameters.

**Fig. 4:**
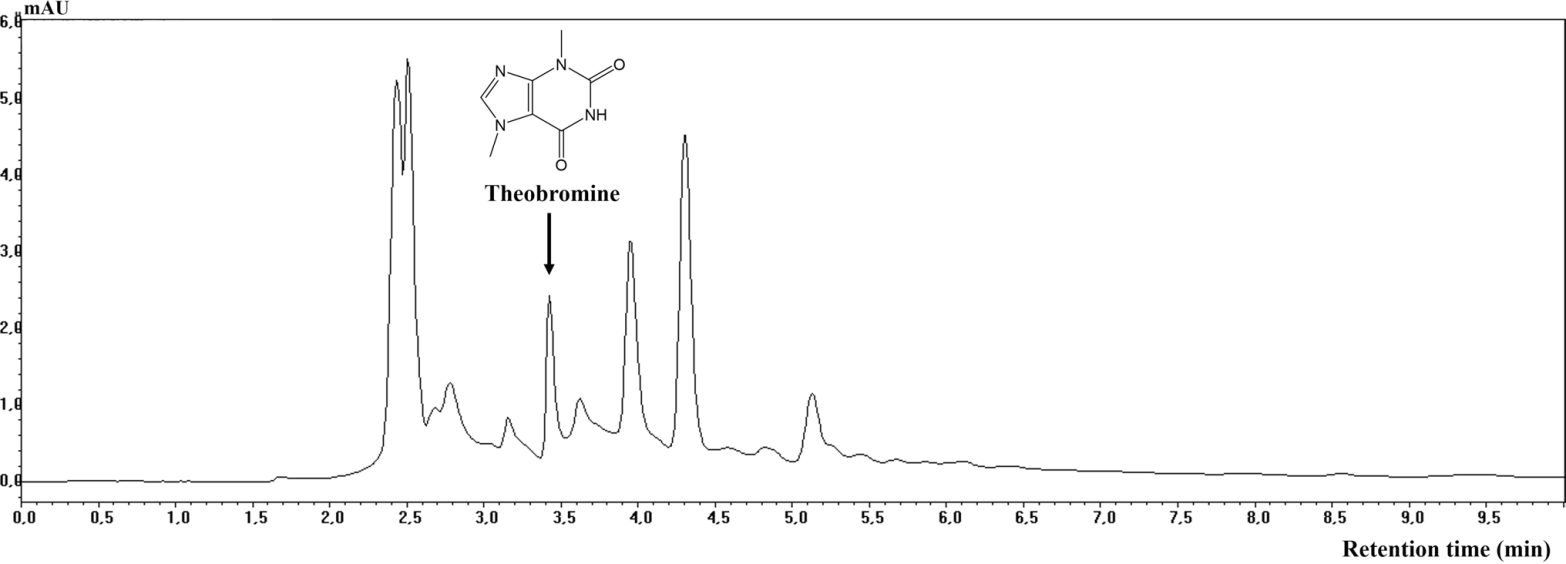
HPLC-DAD chromatogram of cupuassu seed shell extract obtained under optimal conditions using choline chloride–glycerol NADES

The chromatographic profile obtained at optimal extraction parameters is presented at Fig. 4. The literature reports that theobromine, caffeine, and theophylline are the most widespread methylxanthines, naturally present in different plant species, such as cocoa, coffee, tea, yerba mate, and therefore widely present in the human diet [27]. Kandeepan et al. [28] reported that theobromine has several health benefits. Additionally, various techniques have been used for its detection, including ultraviolet spectroscopy, high-performance liquid chromatography, and gas chromatography coupled to mass spectrometry.

Cai et al. [29] optimized the extraction of caffeine from Chinese dark tea by UAE, using various choline-based deep eutectic solvents. Among the tested solvents, choline chloride–lactic acid (ChCl-LA) exhibited the highest extraction yields, suggesting it to be the most efficient system. This can be explained by the assumption that the stronger hydrogen bonding interaction can occur between caffeine and ChCl-LA. Moreover, the authors observed that increasing the concentration of ChCl-LA within the range of 50–70 wt% led to improved extraction efficiency. Additionally, the presence of water in the DES helped reduce viscosity, facilitating mass transfer.

A similar observation was reported by Loukri et al. [13], who found that increasing water content in a eutectic solvent—identical to that used in this study—enhanced by dynamic maceration the caffeine extraction yield from coffee pulp. The high efficiency achieved was attributed to strong multi-interactions between the solvent and target compounds. Additionally, various DESs such as betaine–sorbitol, choline chloride–sorbitol, and their combinations with urea have been investigated for the extraction of caffeine, highlighting the versatility and effectiveness of these green solvents.

Nascimento et al. [14] highlighted that glycerol-based NADES exhibit strong performance in botanical extractions and are particularly effective for the recovery of alkaloids such as methylxanthines, which display favorable solubility and stability in these media. However, the authors reported low caffeine content (2.46 mg g⁻^1^ DM), under optimized conditions by UAE, a trend that remained consistent when using the EDGE system (3.53 mg g⁻^1^ DM) with an ethanol–water mixture [13]. Although *T. cacao* and *T. grandiflorum* belong to the same genus, differences in their metabolic profiles can lead to significant variations in methylxanthine biosynthesis, which may explain the absence of caffeine in the present study.

Moreover, the use of NADES for phytochemical extraction in the EDGE system remains largely underexplored, representing a promising approach for sustainable and efficient bioactive compound recovery. As highlighted by Dos Santos et al. [10], this may be attributed to technical challenges associated with integrating eutectic solvents into the instrument. Despite its potential for green extractions, the efficiency of this approach is highly affected by pH and viscosity. Properly managing these variables is essential to optimize extraction efficiency and expand the applicability of NADES within the EDGE framework.

Therefore, this study is the first to optimize NADES—specifically a choline chloride and glycerol mixture—with the EDGE system, overcoming previously reported drawbacks and offering a more environmentally friendly alternative to traditional organic solvents, while also promoting the valorization of CSS residue through sustainable extraction practices.

## Conclusion

This study highlights the underexplored potential of CSS as a valuable source of bioactive compounds. By applying a dual-extraction strategy aligned with green chemistry and circular economy principles, it was possible to obtain lipids and theobromine using a single, efficient, and environmentally friendly platform. The results not only reinforce the feasibility of valorizing agro-industrial residues, but also demonstrate the relevance of integrating sustainable extraction technologies such as EDGE into bioresource processing. Altogether, this approach contributes to the development of cleaner production chains while adding economic and functional value to an abundant agro-industrial byproduct.

## Data Availability

All data is available from the corresponding author upon reasonable request.

## Abbreviations

ANOVA: Analysis of variance
ATR-FTIR: Attenuated total reflectance Fourier transform infrared spectroscopy
BBD: Box-Behnken design
CS: Cupuassu seed
CSS: Cupuassu seed shell
DSPE: Dispersive solid-phase extraction
EDGE: Energized dispersive guided extraction
GC-FID: Gas chromatography coupled to flame ionization detector
HBA: Hydrogen bond acceptor
HBD: Hydrogen bond donor
HPLC-DAD: High-performance liquid chromatography coupled to diode array detection
NADES: Natural deep eutectic solvents
PLE: Pressurized liquid extraction
RSM: Response surface methodology
